# Mathematical Model Predicts that Acceleration of Diabetic Wound Healing is Dependent on Spatial Distribution of VEGF-A mRNA (AZD8601)

**DOI:** 10.1007/s12195-021-00678-9

**Published:** 2021-06-15

**Authors:** S. Michaela Rikard, Paul J. Myers, Joachim Almquist, Peter Gennemark, Anthony C. Bruce, Maria Wågberg, Regina Fritsche-Danielson, Kenny M. Hansson, Matthew J. Lazzara, Shayn M. Peirce

**Affiliations:** 1 Department of Biomedical Engineering, University of Virginia, Charlottesville, VA USA; 2 Department of Chemical Engineering, University of Virginia, Charlottesville, VA USA; 3 Robert M. Berne Cardiovascular Research Center, University of Virginia, Charlottesville, VA USA; 4 Bioscience Cardiovascular, Research and Early Development, Cardiovascular, Renal and Metabolism, BioPharmaceuticals R&D, AstraZeneca, Gothenburg, Sweden; 5 Drug Metabolism and Pharmacokinetics, Research and Early Development, Cardiovascular, Renal and Metabolism, BioPharmaceuticals R&D, AstraZeneca, Gothenburg, Sweden; 6 Fraunhofer-Chalmers Centre, Chalmers Science Park, Gothenburg, Sweden; 7 Clinical Pharmacology and Quantitative Pharmacology, Clinical Pharmacology and Safety Sciences, R&D, AstraZeneca, Gothenburg, Sweden; 8 Department of Biomedical Engineering, Linköping University, Linköping, Sweden; 9 Research and Early Development, Cardiovascular, Renal and Metabolism, BioPharmaceuticals R&D, AstraZeneca, Gothenburg, Sweden

**Keywords:** Computational modeling, Drug delivery, Partial differential equations, Chronic wounds, Diabetic ulcers

## Abstract

**Introduction:**

Pharmacologic approaches for promoting angiogenesis have been utilized to accelerate healing of chronic wounds in diabetic patients with varying degrees of success. We hypothesize that the distribution of proangiogenic drugs in the wound area critically impacts the rate of closure of diabetic wounds. To evaluate this hypothesis, we developed a mathematical model that predicts how spatial distribution of VEGF-A produced by delivery of a modified mRNA (AZD8601) accelerates diabetic wound healing.

**Methods:**

We modified a previously published model of cutaneous wound healing based on coupled partial differential equations that describe the density of sprouting capillary tips, chemoattractant concentration, and density of blood vessels in a circular wound. Key model parameters identified by a sensitivity analysis were fit to data obtained from an *in vivo* wound healing study performed in the dorsum of diabetic mice, and a pharmacokinetic model was used to simulate mRNA and VEGF-A distribution following injections with AZD8601. Due to the limited availability of data regarding the spatial distribution of AZD8601 in the wound bed, we performed simulations with perturbations to the location of injections and diffusion coefficient of mRNA to understand the impact of these spatial parameters on wound healing.

**Results:**

When simulating injections delivered at the wound border, the model predicted that injections delivered on day 0 were more effective in accelerating wound healing than injections delivered at later time points. When the location of the injection was varied throughout the wound space, the model predicted that healing could be accelerated by delivering injections a distance of 1–2 mm inside the wound bed when compared to injections delivered on the same day at the wound border. Perturbations to the diffusivity of mRNA predicted that restricting diffusion of mRNA delayed wound healing by creating an accumulation of VEGF-A at the wound border. Alternatively, a high mRNA diffusivity had no effect on wound healing compared to a simulation with vehicle injection due to the rapid loss of mRNA at the wound border to surrounding tissue.

**Conclusions:**

These findings highlight the critical need to consider the location of drug delivery and diffusivity of the drug, parameters not typically explored in pre-clinical experiments, when designing and testing drugs for treating diabetic wounds.

**Supplementary Information:**

The online version contains supplementary material available at 10.1007/s12195-021-00678-9.

## Introduction

Diabetic foot ulcers are a type of chronic wound that can persist for months to years because the normal mechanisms of wound healing are profoundly impaired in diabetic patients. Over 30 million Americans are affected by diabetes, and nearly 15% of these patients experience diabetic foot ulcers in their lifetime.^6^,^30^ Diabetic foot ulcers are the leading cause of hospitalizations for patients with diabetes, and are associated with significant pain, suffering, loss of quality of life, and increased risk for lower extremity amputation.^6^ Current therapies for treating chronic diabetic wounds have limited efficacy, and diabetic wounds remain a costly and challenging clinical problem. The development of new therapies for healing chronic diabetic wounds would have a substantial impact on individual patients and on society, but is challenged by a lack of model systems for designing drug delivery strategies that predict the influences of dosages, delivery routes and locations, and mechanisms of action. A novel approach that has been shown to accelerate angiogenesis and the healing of cutaneous wounds in a murine model of diabetic wound healing is the delivery of a modified mRNA (AZD8601) designed to enhance VEGF-A expression in the skin.^38^ There is limited spatially resolved data available about the diffusion and degradation of this modified mRNA and a limited number of drug delivery parameters that have been tested in preclinical models. To address this challenge, we developed a mathematical model that predicts how location of delivery and spatial distribution of AZD8601 impact the rate of wound closure in an established murine model of diabetic wound healing.

Wound healing is a complex and coordinated series of cellular and molecular events comprised of coagulation, inflammation, granulation tissue formation, angiogenesis, reepithelization and extracellular matrix remodeling.^10^ During acute wound healing in healthy individuals, cells respond dynamically to chemotactic cues (e.g., inflammatory cytokines and growth factors) to coordinate this cascade of events, which eventually leads to complete wound closure. However, this coordinated progression is dysregulated in the case of chronic wounds. In particular, patients with diabetes experience microvascular dysfunction^10^ combined with low grade chronic inflammation,^31^ which delays or prohibits the normal wound healing process via a peak in inflammation that triggers the proliferative phase.

For decades, numerous pharmacological treatments designed to promote effective wound healing in diabetic patients have been evaluated in pre-clinical and clinical studies.^12^,^13^,^21^ Stimulation of angiogenesis, or new microvessel formation,^3^,^41^ has been pursued as one approach to jump-start the wound healing cascade in the angiogenesis-impaired setting of diabetes. For example, delivery of both recombinant VEGF-A protein and naked or adenoviral vector-mediated gene transfer to upregulate VEGF-A have been shown to accelerate wound healing in pre-clinical^7^,^19^ and clinical studies.^17^ Our team has recently shown that intradermal injection of AZD8601, an mRNA designed to upregulate VEGF-A expression, accelerates angiogenesis and the healing of cutaneous wounds without causing edema or micro-hemangioma formation in an established murine model of diabetic wound healing.^38^ While these results were encouraging, the reproducibility and extensibility of our experiments, like most pre-clinical studies in the diabetic wound healing field, are challenged and constrained by the fact that only a limited number of drug dosages, drug injection timings, and spatial locations of intradermal injections were tested *in vivo*, allowing for the possibility that a more effective injection protocol was not evaluated.

Mathematical and computational models have demonstrated utility in leveraging experimental data to predict the outcomes of hypothetical experiments that have not yet been tested at the bench.^28^ Running *in silico* experiments using computational and mathematical modeling can also save time, money, and reduce the number of animals needed for experimentation. Previous computational models have been developed to study mechanisms of cutaneous wound healing^2^,^14^,^22^,^32^,^34^,^37^ and to identify drug targets for stimulating angiogenesis in wound healing.^23^ Almquist *et al*. recently reported an empirical pharmacokinetic and pharmacodynamic model of AZD8601 in diabetic wound healing, which captures statistical variation in wound healing dynamics at both the individual and the population level using a nonlinear mixed effect (NLME) modelling approach.^1^ While this model describes the time-dependent aspects of wound healing, it does not account for spatial heterogeneity of drug delivery and wound healing. Therefore, we modified a previously published partial differential equation (PDE) model of cutaneous wound healing originally reported by Pettet *et al*.^29^ that describes the spatiotemporal regulation of chemoattractant production, capillary tip sprouting, and neovascularization to also include a PDE that describes the spatiotemporal dynamics of mRNA and VEGF-A production following injections of AZD8601. We then used this new system of coupled PDEs to predict how diffusivity of mRNA and location of AZD8601 injection in the wound affect angiogenic sprouting, vascularization of the wound bed, and time to wound closure in a model of diabetic wound healing.

## Materials and Methods

### Murine Model of Diabetic Wound Healing

**Figure 1:**
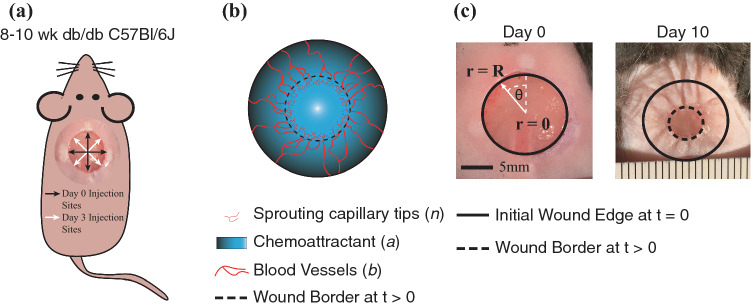
Experimental model and PDE model geometry. (**a**) Full thickness cutaneous wounds of approximately 1 cm in diameter were made on the dorsum of db/db mice and injected with AZD8601 at four injection sites at the initial wound edge separated by 90°. (**b**) The PDE model describes the density of sprouting capillary tips (*n*), chemoattractant concentration (*a*), and blood vessel density (*b*) in a healing wound. (**c**) Model geometry in cylindrical coordinates depicting uniform circular healing. At *t* = 0, the initial wound edge is at *r* = R (5 mm), and healing occurs towards *r* = 0. Healing occurs uniformly with respect to *θ*. Examples of a wound on the dorsum of a mouse are shown on day 0 and day 10.

All procedures were conducted in accordance with the guidelines of the University of Virginia Animal Care and Use Committee or the Local Ethics Committee on Animal Experiments in Gothenburg, Sweden. Three different experimental studies were carried out in two different research laboratories: (1) in the Biomedical Engineering Department at the University of Virginia, Charlottesville, VA, USA, and (2) in the Bioscience Cardiovascular Department, Research and Early Development, Cardiovascular, Renal and Metabolism, BioPharmaceuticals R&D, AstraZeneca, Gothenburg, Sweden. The data acquired in these experiments have been published, along with detailed experimental methods by Sun *et al*.^38^ and Almquist *et al*.^1^ Briefly, circular, full-thickness, cutaneous wounds approximately 1 cm in diameter were surgically made on the dorsum of anesthetized eight-week old diabetic B6.BKS(D)-Lepr^db^/J mice (Jackson Laboratory). Mice received injections of either vehicle or AZD8601 in 10 *µ*L of 10 mM citrate/130 mM saline intradermally at four equidistant points around the wound edge. Some groups of mice received injections at a single timepoint (on day 0 or day 3), and some groups received injections on multiple days (days 0 and 3). For the study groups that were injected twice, the four injection sites were shifted 45° on day 3 in order to avoid injecting the same location twice (Fig. 1a). Wounds in anesthetized mice were serially imaged using an iPhone6 (Apple) or a Canon 600D with a Tamron SP 900 mm F/2.8 objective under bright-field illumination. The open wound area, identified as the region in the center of the wound lacking an epithelial layer, was measured by tracing the border of the wound in ImageJ (Schneider *et al*. 2012). The study groups and time points at which images were acquired and quantified for each study are provided in Table 1. In total, 584 open wound area measurements were made. Percent open wound area was calculated by dividing the wound area measurement at each time point by the initial wound area (at *t* = 0 days) for individual animals. It should be noted that some wounds increased in size by day 3 due to initial wound recoil, leading to wound area measurements at day 3 that were greater than the initial area for some individual animals.

**Table 1:** Summary of experimental design across all studies.

	Location	Study groups	Treatment	Time points measured
Study 1	University of Virginia	Group 1	Vehicle Day 0	Days 0, 3, 6, 10, 13, and 18
		Group 2	100 *µ*g AZD8601 Day 0	
		Group 3	Vehicle Days 0 and 3	
		Group 4	100 *µ*g AZD8601 Days 0 and 3	
Study 2	University of Virginia	Group 1	Vehicle Days 0 and 3	Days 0, 3, 6, 10, and 13
		Group 2	30 *µ*g AZD8601 Days 0 and 3	
		Group 3	100 *µ*g AZD8601 Days 0 and 3	
		Group 4	200 *µ*g AZD8601 Days 0 and 3	
Study 3	AstraZeneca, Sweden	Group 1	Vehicle Day 3	Days 0, 3, 7, 10, 14, and 17
		Group 2	100 *µ*g AZD8601 Day 3	
		Group 3	Vehicle Days 0 and 3	
		Group 4	100 *µ*g AZD8601 Days 0 and 3	

### Pharmacokinetic Model

In a separate study, the pharmacokinetics (PK) of VEGF-A protein was measured after injection of a single dose of 100 *µ*g AZD8601 intradermally in mice without wounds. Briefly, intradermal injections were given in up to three different locations (*n* = 13 mice). Mice were sacrificed at 6, 24, 48, 72, and 144 h after injection, and the amount of VEGF-A protein in the skin was quantified according to methods that have been published previously.^38^ The lower limit of quantification (LLOQ) for VEGF-A was 0.156 pg/mg tissue. At 144 h, 10 out of 12 observations were below LLOQ. This study, including the bioanalytical methods for quantifying the amount of VEGF-A protein in skin, is described in detail by Sun *et al*.^38^

The PK of AZD8601 (VEGF-A mRNA) and VEGF-A protein was represented by the following model:

1 $$\frac{{{\text{dmRNA}}\left( t \right)}}{{{\text{d}}t}} = - k_{1} \times {\text{mRNA}}\left( t \right)$$

2 $$\frac{{{\text{dVEGF}}\left( t \right)}}{{{\text{d}}t}} = k_{2} \times {\text{mRNA}}\left( t \right) - k_{3} \times {\text{VEGF}}\left( t \right)$$

3 $${\text{mRNA}}\left( 0 \right) = {\text{VEGF}}\left( 0 \right) = 0$$

where mRNA(t) is the amount of AZD8601 and VEGF(t) is the concentration of VEGF-A protein. The parameters *k*_1_, *k*_2_, and *k*_3_ are kinetic parameters describing degradation of mRNA, synthesis of VEGF-A, and degradation of VEGF-A, respectively (Table 2). Note that these parameters are only valid for full-thickness wounds of 1 cm in diameter. A complete description of this model and its underlying data is reported by Almquist *et al*.^1^

**Table 2:** Pharmacokinetic model parameters.

Description	Symbol	Unit	Value
mRNA degradation	*k*_1_	h^−1^	0.055
VEGF synthesis	*k*_2_	pg (VEGF-A) mg^−1^ (tissue) *μ*g^−1^ (mRNA) h^−1^	0.16
VEGF degradation	*k*_3_	h^−1^	0.23

### Partial Differential Equation Model

A PDE model was implemented in MATLAB (R2020a, The MathWorks, Natick, MA) based on the wound healing model previously described by Pettet *et al*.^29^ This model treats the wound as a one-dimensional geometry with healing occurring in one direction, from the edge to the center of the wound. The system of coupled PDEs (Eqs. 4–6) published by Pettet *et al*. describes three dependent variables: sprouting capillary-tip density (*n*), chemoattractant concentration (*a*), and blood vessel density (*b*). A schematic of these variables in a healing wound is illustrated in Fig. 1b. The dimensionless conservation equations from Pettet *et al*. in Cartesian coordinates are given by:

4 $$\frac{\partial n}{\partial t} = \mu_{n} \frac{{\partial^{2} n}}{{\partial x^{2} }} - \chi \frac{\partial }{\partial x}\left( {n\frac{\partial a}{\partial x}} \right) + \lambda_{1} ab - \lambda_{2} n - \lambda_{0} n^{2}$$

5 $$\frac{\partial a}{\partial t} = \frac{{\partial^{2} a}}{{\partial x^{2} }} + \frac{{\lambda_{4} }}{2}\left( {1 + \tanh \left( {\frac{1 - b}{\delta }} \right)} \right) - \left( {\lambda_{4} + \lambda_{5} b} \right)a$$

6 $$\frac{\partial b}{\partial t} = \mu_{b} \frac{\partial }{\partial x}\left( {n\frac{\partial b}{\partial x}} \right) - \mu_{n} \frac{\partial n}{\partial x} + \chi n\frac{\partial a}{\partial x}$$

Descriptions and values for the constant parameters in these equations are given in Table 3. In this model, chemoattractant is defined broadly to represent proangiogenic factors secreted by macrophages that promote migration of sprouting endothelial cells and wound healing. Macrophages are assumed to be evenly distributed throughout the wound space. The chemoattractant profile drives the wave-like ingrowth of capillary-tip sprouts and new blood vessel formation, representing angiogenesis that occurs during wound healing. The formation and chemotaxis of sprouting endothelial cells from the existing vasculature occurs via both random motility of sprouts and migration up the chemotactic gradient. The kinetic terms associated with sprouting capillary-tip density include production via budding and loss of capillary-tips due to decay and tip-to-tip anastomosis (Eq. 4). The equation for chemoattractant concentration (Eq. 5) describes the diffusion of chemoattractant, the production by macrophages in the wound space, removal via the vasculature, and decay of chemoattractant. The blood vessel density (Eq. 6) is described primarily by the maturation of migrating capillaries connected with the existing vasculature and random motility of the capillary tips. Full details for the original system of equations are described by Pettet *et al*.

**Table 3:** PDE model parameters in Cartesian and cylindrical coordinate systems.

Parameter	Parameter description	Parameter value in Cartesian coordinates described by Pettet et al. (dimensionless)	Parameter value in cylindrical coordinates (dimensionless) [fitted value]
*λ*_0_	Rate of tip anastomosis	100	200 [856]
*λ*_1_	Tip production	100	800 [3860]
*λ*_2_	Decay of sprouting tips	10	40
*μ*_n_	Capillary tip coefficient of random motility	10^−3^	10^−3^
*χ*	Capillary tip coefficient of chemotaxis	0.1	0.1 [0.146]
$\hat{n}$	Tip density at wound edge	1	2
*α*	Rate of decay of tip density	2.5	10
*λ*_4_	Decay of chemoattractant concentration	100	400
*λ*_5_	Removal of chemoattractant via vasculature	10	40
*λ*_7_	Constant rate of blood-borne removal	10	20
*δ*	Rate of decreasing chemoattractant production	0.01	0.01
$\mu_{b}$	Blood vessel coefficient of random motility	10^−3^	0.5 × 10^−3^
$\hat{b}$	Blood vessel density at wound edge	1.5	1.5
$\tilde{r}$	Margin around wound edge that delineates inflammation zone in initial response	0.05	0.95

### Conversion to Cylindrical Coordinates

We converted the equations described by Pettet et al. from Cartesian to cylindrical coordinates to represent the geometry of the wound more accurately. In the converted model, the wound is circular with an initial radius *r* = *R*, placing the center of the wound at *r* = 0, with healing occurring in the negative *r* direction (Fig. 1c). The governing equations in Cartesian form (Eqs. 4–6) were rewritten in general differential form and then recast in cylindrical coordinates. The characteristic length in the model described by Pettet *et al*. is *L* = 2.5 mm, whereas our model is defined with a radius *R* = 5 mm to reflect the conditions of our experimental wound healing model. Thus, in our model, the radial coordinate (*r*) is non-dimensionalized by *R* = 5 mm and the time coordinate (*t*) is non-dimensionalized by *R*^2^/*D* = 2.89 days, considering a representative chemoattractant diffusivity of *D* = 10^−6^ cm^2^/s based on the diffusivity of acidic fibroblast growth factor in agarose.^35^ Additionally, all dimensionless parameters that appear in Eqs. 4–6 retain their original definitions (found between Eqs. 9 and 10 in Pettet *et al*.) in the new set of cylindrical model equations, except that the wound half-width (L) from the original definitions has been replaced by the wound radius (R) in our system. A detailed explanation of the model transformation from Cartesian to cylindrical coordinates is described in the supplemental methods (Sections S1 and S2).

Because the wounds in the murine models are full-thickness, we assume that no healing occurs in the *z*-direction. Additionally, because the wound diameter (~1 cm) is much greater than the wound thickness, we assume that the field variables can be lumped in the *z*-direction and therefore modeled solely in the r-direction. This results in a new set of dimensionless conservation equations for *n*, *a*, and *b*:

7 $$\frac{\partial n}{\partial t} = \mu_{n} \frac{1}{r}\frac{\partial }{\partial r}\left( {r\frac{\partial n}{\partial r}} \right) - \chi\, n\frac{1}{r}\frac{\partial }{\partial r}\left( {r\frac{\partial a}{\partial r}} \right) - \chi \frac{\partial a}{\partial r}\frac{\partial n}{\partial r} + \lambda_{1} ab - \lambda_{2} n - \lambda_{0} n^{2}$$

8 $$\frac{\partial a}{\partial t} = \frac{1}{r}\frac{\partial }{\partial r}\left( {r\frac{\partial a}{\partial r}} \right) + \frac{{\lambda_{4} }}{2}\left[ {1 + \tanh \left( {\frac{1 - b}{\delta }} \right)} \right] - \bigg(\lambda_{4} + \lambda_{5} b\bigg)a$$

9 $$\frac{\partial b}{\partial t} = \mu_{n} \left[ {n\frac{1}{r}\frac{\partial }{\partial r}\left( {r\frac{\partial b}{\partial r}} \right) + \frac{\partial b}{\partial r}\frac{\partial n}{\partial r}} \right] - \mu_{n} \frac{\partial n}{\partial r} + \chi \,n\frac{\partial a}{\partial r}$$

The dimensionless initial conditions in cylindrical coordinates are given by:

10 $$n\left( {r,0} \right) = \left\{ \begin{array}{ll} \frac{{\hat{n}}}{{\tilde{r}^{3} }}\left( {r - \tilde{r}} \right)\left( {2r^{2} - \tilde{r}r - \tilde{r}^{2} } \right), & \tilde{r} < r \le 1 \\ 0, & 0 \le r \le \tilde{r} \\ \end{array} \right.$$

11 $$a\left( {r,0} \right) = 0, \quad 0 \le r \le 1$$

12 $$b\left( {r,0} \right) = \left\{ {\begin{array}{ll} {\left( {\frac{{\hat{b} - \tilde{b}}}{{\tilde{r}^{3} }}} \right)\left( {r - \tilde{r}} \right)\left( {2r^{2} - \tilde{r}r - \tilde{r}^{2} } \right) + b,} & {\tilde{r} < r \le 1} \\ {\tilde{b},} & {0 \le r \le \tilde{r}} \\ \end{array} } \right.$$

where it is assumed that the wound margin has penetrated an initial distance 1 − $\tilde{r}$ such that the radius of the open wound in dimensionless terms is $\tilde{r}$. We note that the initial conditions for *n* and *b* (Eqs. 10 and 12) are those reported by Pettet et al. and were justified by their monotonic behavior in the inflammation zone. The dimensionless boundary conditions in cylindrical coordinates are given by:

13 $$\frac{\partial n}{\partial r}\left( {0,t} \right) = 0$$

14 $$n\left( {1,t} \right) = \hat{n}e^{ - \alpha t}$$

15 $$\frac{\partial a}{\partial r}\left( {0,t} \right) = 0$$

16 $$\frac{\partial a}{\partial r}\left( {1,t} \right) = - \lambda_{7} a\left( {1,t} \right)\hat{b}$$

17 $$\frac{\partial b}{\partial r}\left( {0,t} \right) = 0$$

18 $$b\left( {1,t} \right) = \hat{b}$$

where $\hat{n}$ is the capillary tip density at the wound edge; *α* is rate of decay of tip density; *λ*_*7*_ is the rate of removal of chemoattractant *via* vasculature; and $\hat{b}$ is the blood vessel density at the wound edge. The model equations were solved using an explicit finite difference method, as described in the supplemental methods (Section S3).

Since wound area is not a direct output of the PDE model, in order to compare model predictions to experimental measurements of wound area, the percent open wound area in the PDE model was determined by the area where the blood vessel density (*b*) was less than 0.1 (dimensionless units) divided by the initial wound area ($\pi R^{2}$). This is intended to represent the border of granulation tissue, composed of neovessels, which fills in the wound during healing and was quantified in experimental studies (Figs. 1b and 1c).

### Sensitivity Analysis and Parameter Fitting

A sensitivity analysis was performed to determine the influence of each of the 13 model parameters (Table 3) on the rate of wound healing, as described by the percent open wound area. Each parameter was increased and decreased by 10%, varying only one parameter at a time, and a simulation using the perturbed parameters was compared to a simulation using the unperturbed parameters. Specifically, a sensitivity coefficient, *S*, was calculated using

19 $$S = \frac{{y_{i} - y_{o} }}{{|p_{i} - p_{o} |}} \times \frac{{p_{o} }}{{y_{o} }}$$

where *y*_*o*_ and *y*_*i*_ are the measured percent open wound area at *t* = 18 days when parameters are set to baseline or perturbed levels, respectively, and *p*_*o*_ and *p*_*i*_ are the values of the baseline parameter and perturbed parameter, respectively. By measuring the absolute difference between the baseline and perturbed parameter, the sign of the sensitivity coefficient, *S*, can be interpreted as the direction of change in the measured output. Therefore, a positive sensitivity coefficient would indicate an increase in percent open wound area at *t* = 18 days, and a negative sensitivity coefficient would indicate a decrease in percent open wound area. Additionally, the sensitivity coefficient is normalized by the baseline parameter value and wound area to account for order of magnitude variations in parameter values.

Subsequent parameter fitting was performed using an optimization function (the *particleswarm* function with default settings in MATLAB, R2020a, The MathWorks, Natick, MA) to search the parameter space for a combination of parameter values that minimize the objective function. The objective function used was the sum of squared errors (SSE) between experimental measurements of wound area and model simulated wound area at the specified experimental time points. The parameter space was constrained by lower and upper bounds of 0.1-fold and 10-fold changes from the baseline value to maintain parameter values within physiologically plausible ranges.

### Simulating Injections of AZD8601 with the PDE Model

Injections of AZD8601 were modeled by a PDE to describe the spatiotemporal distribution of mRNA in the wound space, which was then coupled to the equation for chemoattractant (Eq. 8) to describe VEGF-A synthesis as a function of the local mRNA concentration. In dimensionless form, the conservation equation for mRNA is given by

20 $$\frac{\partial m}{\partial t} = \frac{{D_{m} }}{{D_{a} }}\frac{1}{r}\frac{\partial }{\partial r}\left( {r\frac{\partial m}{\partial r}} \right) - \left( {\frac{{R^{2} }}{{D_{a} }}} \right)k_{1} m$$

where *m* is the dimensionless concentration of mRNA, *D*_*m*_ is the diffusivity of mRNA, *D*_*a*_ is the diffusivity of chemoattractant, and *k*_1_ is the first-order rate constant of mRNA degradation as defined above by the PK model. We set *D*_*a*_ to be the typical diffusivity of chemoattractant (10^−6^ cm^2^/s) as previously reported by Pettet *et al*.^29^,^35^ Reported values in literature for the diffusivity of mRNA are in the range of 2 × 10^−9^ − 4 × 10^−9^ cm^2^/s,^5^,^18^,^43^ so we set mRNA diffusivity (*D*_m_) to be 3 × 10^−9^ cm^2^/s. In Eq. 20, *m* has been scaled and non-dimensionalized using the concentration of 100 ug mRNA in a total injection volume of 40 *µ*L (2500 *µ*g/cm^3^), as described in the murine experimental model. Note that the quantity $\left( {\frac{{R^{2} }}{{D_{a} }}} \right)$ is required to non-dimensionalize *k*_*1*_. A no-flux boundary condition is imposed at the wound center:

21 $$\frac{\partial m}{\partial r}\left( {0,t} \right) = 0$$

and clearance of mRNA at the wound edge is described by the Robin boundary condition

22 $$\frac{\partial m}{\partial r}(1,t) = - \lambda_{8} \hat{b}\,m(1,t)$$

where $\lambda_{8}$ is the dimensionless rate constant of mRNA removal via vasculature at the wound edge. We assume that this rate of clearance by the vasculature is comparable for chemoattractant and mRNA, so we set $\lambda_{8}$ = $\lambda_{7}$ (the rate of blood-borne removal for chemoattractant).

The governing equation for chemoattractant is modified to include an mRNA-dependent chemoattractant generation term to yield, in dimensionless form,

23 $$\frac{\partial a}{\partial t} = \frac{1}{r}\frac{\partial }{\partial r}\left( {r\frac{\partial a}{\partial r}} \right) + \frac{{\lambda_{4} }}{2}\left[ {1 + \tanh \left( {\frac{1 - b}{\delta }} \right)} \right] - \bigg( {\lambda_{4} + \lambda_{5} b}\bigg ) \,a + k_{\text{gen}} m$$

where *k*_gen_ is the dimensionless first-order rate constant for VEGF generation. All other variables and parameters are as previously defined. The dimensionless VEGF generation rate constant (*k*_gen_) was fit by minimizing the SSE between the net amount of chemoattractant generated by the PDE model and experimental measurements of VEGF-A following an injection with 100 *μ*g of AZD8601 previously reported by Almquist *et al*.^1^ Since the PK experiments were performed in non-injured tissue, the PDE model was run to steady state to simulate a healed wound and then 100 *μ*g of mRNA was uniformly distributed throughout the wound space. VEGF-A generated by injections with AZD8601 is considered part of the chemoattractant pool in this model implementation. The net amount of generated chemoattractant from the PDE model was calculated by subtracting the total amount of chemoattractant from the unperturbed PDE model (without injections) from the total amount of chemoattractant in a simulation with a single 100 *μ*g injection. The dimensionless fitted value for *k*_gen_ was 1.53 × 10^5^.

## Results

### Sensitivity Analysis Identifies Parameters for Model Fitting

**Figure 2:**
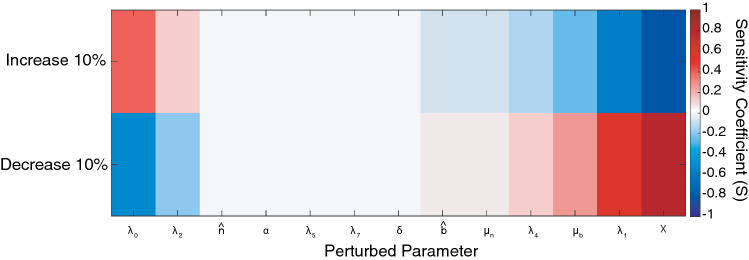
Sensitivity analysis of open wound area with respect to individual model parameters. Sensitivity coefficients (*S*) were calculated when individual parameters were increased or decreased by 10% one at a time while holding all other parameters at baseline values. *S* values around zero correspond to low sensitivity, while positive *S* values indicate an increase in open wound area, and negative *S* values indicate a decrease in open wound area at *t* = 28 days.

The original system of equations described by Pettet *et al*. was based on a rabbit model of wound healing in the ear skin. Our experimental model of wound healing is in the dorsum of mice, which contains a layer of thin muscle, termed the “panniculosus carnosus”, and causes rapid contraction of the wound in the late stages of acute wound healing leading to faster rates of wound closure.^42^ This phenomenon is apparent in the image of a healing wound in a mouse at day 10 (Fig. 1c), as evidenced by the lines of tented skin extending radially outward from the wound. Because of this difference in experimental models, we conducted a sensitivity analysis to identify a set of parameters appropriate for fitting the computational model to the rate of wound healing observed in our experimental murine model of diabetic wound healing (see Methods Section 3.5). Figure 2 depicts the unitless sensitivity coefficient (*S*) when 13 model parameters were increased or decreased by 10% one at a time. Due to the high complexity of the parameter fitting problem and data sparsity, the number of free parameters was limited to three. The parameters with greatest sensitivity coefficients were selected as candidates for parameter fitting, namely *λ*_0_, *λ*_1_ and $\chi$.

**Figure 3:**
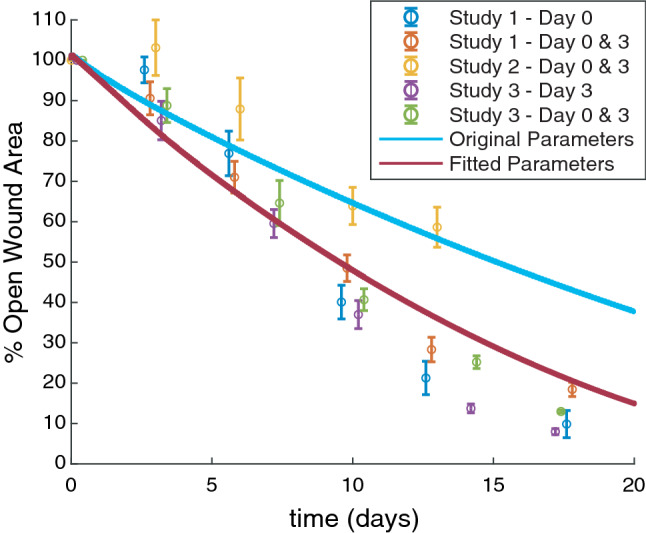
Model parameters were fitted using vehicle data from all experimental studies. The mean of percent open wound area for all animals is plotted as open circles for Study 1 vehicle groups (injected on day 0 or days 0 and 3), Study 2 vehicle groups (injected on days 0 and 3), and Study 3 vehicle groups (injected on day 3 or days 0 and 3), error bars = SEM. Horizontal dodging of up to 0.6 days has been applied to the data so that overlapping data and error bars are more easily visible, but the precise time point of wound area measurement is reported in Table 1. Wound area predicted by the PDE model is shown for a simulation with original parameter values (solid light blue line) and after parameter fitting (solid dark red line).

In order to calibrate the computational model to the basal rate of wound healing in our murine experimental model, we used these selected parameters to fit the PDE model to experimental data from all of the vehicle-injected groups in Studies 1, 2, and 3. The predicted wound areas were compared to experimental measurements of wound area following vehicle injections in all three studies. Parameters were fit by simultaneously varying $\lambda_{0}$, $\lambda_{1}$, and $\chi$ over a range of values constrained by 0.1-fold and 10-fold changes from baseline parameter values, and minimizing the sum of squared error (SSE) between the model predicted wound area and experimental measurements of wound area at all time points for vehicle-injected groups. The fitted values for these dimensionless parameters that minimized the SSE were $\lambda_{0} = 856$, $\lambda_{1} = 3860$, and $\chi = 0.146$, or fold changes from baseline values of 4.3, 4.8, and 1.5, respectively. After parameter fitting, the model generated a time course of healing that was similar to the time course of healing across all vehicle-injected experimental groups (Fig. 3). The experimental data presented here from vehicle-injected groups demonstrates variability in the rate of wound healing between studies. Evaluating the causes and implications of this variability was not the main objective of this study, but is discussed comprehensively in Almquist *et al*.^1^ Notably, Study 2 demonstrated a slower rate of wound closure than Studies 1 and 3 and did not include late time points beyond day 13. Furthermore, considering that Study 2 comprised less than 20% of the data points used for fitting, it is reasonable that parameter fitting resulted in a model output that is in better agreement with Studies 1 and 3.

### PDE Model Describes Spatiotemporal Regulation of Chemoattractant Concentration and Angiogenesis During Cutaneous Wound Healing

**Figure 4:**
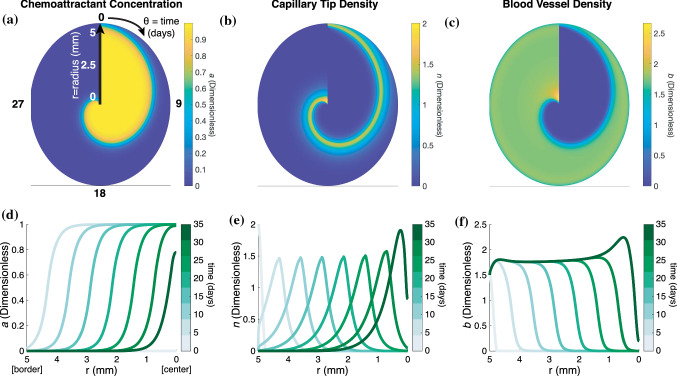
PDE model provides spatial and temporal resolution of wound healing. Heat maps in polar coordinates of the solutions for the governing equations: (**a**) chemoattractant concentration (*a*), (**b**) sprouting capillary-tip density (*n*), and (**c**) blood vessel density (*b*). Radial coordinate corresponds to radius of the wound where *r* = 0 mm is at the center of the wound and *r* = 5 mm at the border of the wound, $\theta$coordinate corresponds to time (0–36 days), and color bar indicates value of the corresponding solution. (**d**–**f**) 2-D snapshots of the heatmaps in **a**-**c** at *t* = 0, 5, 10, 15, 20, 25, 30, and 35 days.

The PDE model provides spatial and temporal information about the dynamics of wound healing with respect to three dependent variables: chemoattractant concentration, sprouting capillary-tip density, and blood vessel density (Figs. 4a–4c). The radial coordinate in these plots represents the radial coordinate of the wounds (spanning 0–5 mm), the azimuthal ($\theta$) coordinate corresponds to time (0–36 days) that increases in a clockwise direction, and the color bar indicates the magnitude of the dependent variables. The chemoattractant profile (Fig. 4d), which is assumed to be produced by macrophages located throughout the wound space, is initially high throughout the wound space and diminishes towards the center of the wound as the wound heals. Figure 4e demonstrates the wave-like ingrowth of capillary-tip sprouts, as was originally described by Pettet *et al*. Intact blood vessels at the edge of the wound extend sprouts that move towards the center of the wound. As the sprouting capillary-tips migrate, they leave in their path a new capillary that matures to become part of the established blood vessel network. The mature blood vessels are able to provide blood and oxygen supply to the healing wound while removing chemoattractant (Fig. 4f). The effects of geometry on the solutions of capillary tip and blood vessel density can be observed at late time points (*t* = 35 days) in the center of the wound (*r* = 0 mm) by comparing our model, which uses a cylindrical coordinate system, to the original solutions reported by Pettet *et al*. The densities of capillary tips and blood vessels increase as they crowd into a smaller wound area and the rate of wound closure slows.

### Pharmacokinetic Model of AZD8601 Injections

**Figure 5:**
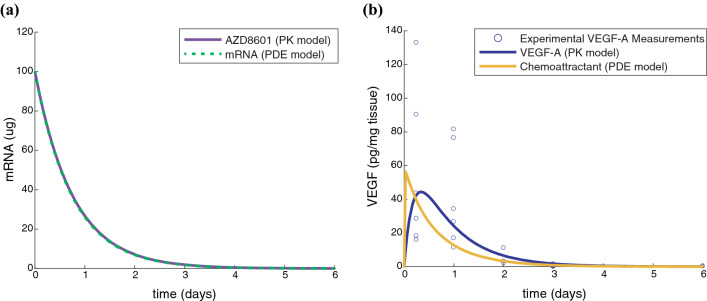
VEGF generation rate is fit using pharmacokinetic model of AZD8601 injections. (**a**) PK model (solid purple line) and PDE model (dashed green line) predict mRNA amount (*μ*g) following injection of 100 *μ*g AZD8601. (**b**) Experimental measurements of VEGF-A concentration were taken at 6, 24, 48, 72, and 144 h after injection with 100 *μ*g AZD8601 (blue circles). PK model (solid blue line) and PDE model (solid yellow line) predict temporal dynamics of VEGF-A concentration (pg/mg tissue) following injection of 100 *μ*g AZD8601.

The PK model published by Almquist *et al*.^1^ was used to describe the degradation of mRNA, as well as synthesis and degradation of VEGF-A protein in the skin following administration of AZD8601. The kinetic parameters (*k*_1_ and *k*_3_) correspond to half-lives of 13 hours and 3 h for degradation of mRNA and protein, respectively. VEGF-A protein levels peaked around 8 h after injection of 100 *μ*g AZD8601, and by day 6, ten of twelve measurements were below the lower limit of quantification. Due to the lack of available data about the spatial parameters of AZD8601 diffusion and clearance rates, we fit a VEGF generation rate (*k*_gen_) based on the PK model-predicted time course of VEGF-A synthesis following a single injection with 100 *μ*g AZD8601. After parameter fitting, the PDE model predicted mRNA and chemoattractant time courses that were consistent with the PK model predictions and experimental measurements of VEGF-A (Fig. 5). However, the PDE model predicted a nearly instantaneous increase in VEGF-A following the addition of mRNA unlike the PK model output which peaks around 8 h (Fig. 5b). This is likely because the PDE model does not account for the time required for cells to uptake the mRNA and begin protein production. It has been shown previously that VEGF-A can be detected in the eluates from interstitial microdialysis sampling approximately 4 h following intradermal injection of AZD8601 in rabbits.^27^

### Modeling the Spatiotemporal Distribution of mRNA Following Injections with AZD8601 Predicts Varied Effects on Wound Healing That Are Dependent on the Timing of Injections

An additional PDE was coupled to the system of equations originally described by Pettet et al. to simulate injections of AZD8601. The spatial parameters of AZD8601 including effective diffusion length scales and clearance rates are not well understood at this time. However, the PK model reported by Almquist *et al*.^1^ describes the temporal dynamics of mRNA and VEGF-A following an injection. We used the PK model to fit a VEGF generation rate that produced a spatially averaged concentration of VEGF-A in the PDE model consistent with the temporal dynamics of mRNA and VEGF-A predicted by the PK model. This model approach allowed us to investigate the impact of parameters related to the spatial distribution of AZD8601 on the rate of wound healing, and simulate experimental conditions that were not tested *in vivo*.

**Figure 6:**
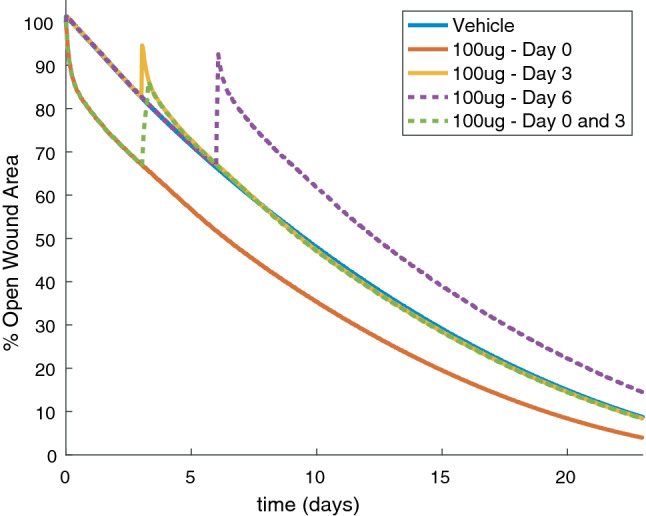
Wound closure is dependent on time of delivery of AZD8601 when injected at the wound border. Percent open wound area over time is presented for PDE model simulations with a vehicle injection (solid blue line), or injections of 100 *μ*g AZD8601 on day 0 (solid red line), day 3 (solid yellow line), day 6 (dashed purple line), and days 0 and 3 (dashed green line).

The mRNA delivered by injections of AZD8601 at the wound border diffuses into the wound based on the reaction-diffusion parameters of the governing equation (Eq. 20) and is translated to VEGF-A at a rate dependent on the local concentration of mRNA (Eq. 23). This model predicted varied effects on the rate of wound healing that were dependent on the time of delivery of the injection (Fig. 6). Simulation of a single injection of 100 *μ*g AZD8601 on day 0 was predicted to accelerate time to 50% wound closure by 3.1 days. Both a single injection of 100 *μ*g AZD8601 on day 3, or repeated injections on days 0 and 3, were predicted to have no significant impact on time to 50% wound closure and instead caused a temporary reversal of blood vessel growth towards the initial wound border. A single injection of 100 *μ*g AZD8601 on day 6 was predicted to delay time to 50% wound closure by 2.9 days.

**Figure 7:**
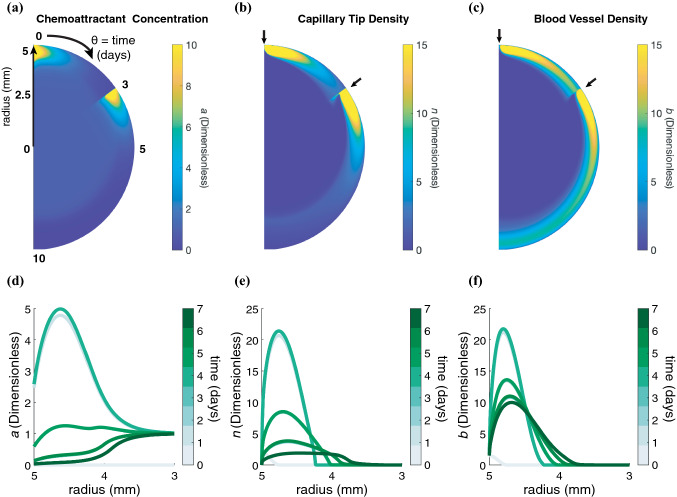
Repeated injections of AZD8601 on days 0 and 3 cause an increase in density of capillary tips and blood vessels at the wound border. Heat maps in polar coordinates of the solutions for the governing equations: (**a**) chemoattractant concentration (*a*), (**b**) sprouting capillary-tip density (*n*), and (**c**) blood vessel density (*b*) for a simulation with repeated injections of 100 *μ*g AZD8601 on days 0 and 3 (indicated by arrows). Radial coordinate corresponds to radius of the wound (0–5 mm), $\theta$coordinate corresponds to time (0–10 days), and color bar indicates value of the corresponding solution. (**d**–**f**) 2-D snapshots of the heatmaps in C-E at *t* = 0, 1, 2, 3, 4, 5, 6 and 7 days at the wound border (*r* = 3–5 mm).

Heat maps of the solution to the governing equations of the PDE model depict the profiles for *a* (Fig. 7a), *n* (Fig. 7b), and *b* (Fig. 7c) for a simulation with repeated injections of 100 *μ*g AZD8601 on days 0 and 3. The chemoattractant profile (Figs. 7a and 7d) shows that the chemoattractant concentration peaks at a distance of approximately 0.4 mm inside the wound border and returns to baseline values approaching the wound center. This results in a peak in capillary tip density at a similar distance inside the wound border following injections with AZD8601 (Figs. 7b and 7e). Injections of AZD8601 at the wound border on day 0 cause an increase in the rate of capillary tip migration towards the center of the wound; however, a second injection on day 3 causes an accumulation of capillary tips and blood vessels at the wound border, preventing the migration of capillary tips towards the center of the wound (Figs. 7c and 7f). The injection on day 3 results in an increase in the density of blood vessels near the wound border that persists throughout the time course of wound healing.

The diffusion and degradation kinetics of mRNA delivered at the wound border cause the VEGF-A concentration to peak a short distance inside the wound border and then return to baseline towards the center of the wound. The resulting gradient of VEGF-A can accelerate the rate of wound closure when delivered on day 0. However, this causes a regression of the blood vessel network towards the wound border and even delays wound closure when an injection is delivered on day 6 compared to a simulation with no injection. At these later time points the border of the blood vessel network has migrated a distance into the wound space that is closer to the center of the wound than the peak VEGF-A concentration created by an injection at the wound border. Thus, this model predicts an effect on wound healing that is dependent on the timing of the injection and spatial distribution of the chemoattractant gradient with respect to the border of the healed blood vessel network at the time of injection.

### Location of AZD8601 Injections Impacts Rate of Wound Healing

**Figure 8:**
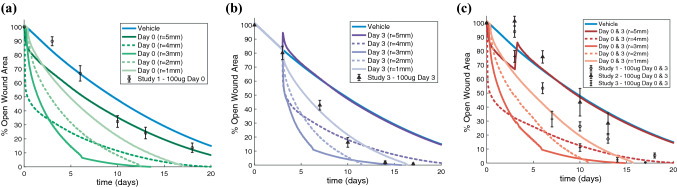
Delivery of AZD8601 at various depths in the wound space impacts rate of wound healing. Percent open wound area is predicted for simulations with injections of 100 *μ*g AZD8601 at locations ranging from the border of the wound (*r* = 5 mm) to near the center of the wound (*r* = 1 mm) for injections delivered on (**a**) day 0, (**b**) day 3, or (**c**) days 0 and 3. These simulations are compared to a simulation with a vehicle injection (solid blue line) and experimental measurements of wound area from a murine wound healing model where injections of 100 *μ*g AZD8601 were delivered at the wound border (*r* = 5 mm) on (**a**) day 0, (**b**) day 3, and (**c**) days 0 and 3. Error bars = SEM.

We used this model to predict the effects of varying the location of injections within the wound space, something that was not tested experimentally. Simulating injections of AZD8601 delivered on day 0 at various locations ranging from the border of the wound (*r* = 5 mm) to the center of the wound (*r* = 1 mm) substantially impacted the rate of wound closure (Fig. 8a). When injections on day 0 were delivered at the wound border (*r* = 5 mm), time to 50% wound closure was predicted to be accelerated by 3 days compared to no injections. The rate of wound closure was predicted to be dramatically accelerated by delivering injections of AZD8601 a short distance inside the wound border on day 0. The maximum impact on time to 50% wound closure was observed at *r* = 4 mm, but this effect was diminished at locations closer to the center of the wound (*r* = 3 mm, 2 mm, and 1 mm). However, the simulation with an injection at *r* = 4 mm predicted a longer time to 100% wound closure than the simulations with an injection delivered further into the wound space. An injection delivered at *r* = 4 mm is predicted to dramatically increase the rate of wound closure at early time points, but this effect is not sustained throughout the time course of wound healing, whereas injections delivered further in the wound space demonstrate a more consistent rate of wound closure. Model predictions were compared with experimental measurements of wound area for animals that received 100 *μ*g injections of AZD8601 on day 0 at the wound border (*r* = 5 mm) (Fig. 8a) and showed close agreement with experimental data at late time points (days 10, 13, and 18), but discrepancies at early time points (days 3 and 6) when compared to a simulated injection at the wound border (*r* = 5 mm).

Simulations were repeated for injections of 100 *μ*g AZD8602 on day 3 at various distances from the wound center (Fig. 8b). An injection at the wound border (*r* = 5 mm) on day 3 was predicted to cause a temporary regression in blood vessel density and wound area, but had no measurable impact on the time to 50% wound closure. However, injections delivered further from the wound border (*r* = 4 mm, 3 mm, and 2 mm) were all predicted to accelerate the time to 50% wound closure by approximately 6 days. This effect was slightly diminished by delivering injections near the center of the wound (*r* = 1 mm), where time to 50% wound closure was predicted to be accelerated by 4 days compared to a simulation with no injections. Model predictions were compared with experimental measurements of wound area for animals that received 100 *μ*g injections of AZD8601 on day 3 at the wound border (Fig. 8b) and demonstrated the most agreement with a simulated injection near the center of the wound (*r* = 1 mm).

We also simulated the impacts of varying the location of injections with repeated injections on days 0 and 3 (Fig. 8c). When injections were delivered at the wound border (*r* = 5 mm) wound healing was accelerated at early time points, but the second injection on day 3 caused a regression of the blood vessels and wound area that resulted in no significant difference in time to 50% wound closure compared to a simulation with a vehicle injection. Similar to simulations with injections on day 0 or 3, an injection delivered at *r* = 4 mm was predicted to significantly increase wound closure at early time points, but that rate of wound healing was not sustained through later time points. Thus, an injection at *r* = 4 mm was predicted to result in the fastest time to 50% wound closure, but injections further in the wound space (*r* = 3 mm, *r* = 2 mm, and *r* = 1 mm) were predicted to have faster times to 100% wound closure. Model predictions were compared to experimental measurements of wound area for animals from experimental groups in three separate studies that received 100 *μ*g injections of AZD8601 on days 0 and 3 at the wound border (Fig. 8c). Experimental data reveal study to study variation in the rate of wound healing, but demonstrated the closest agreement with a simulated injection closer to the center of the wound (*r* = 1 mm) at late time points.

Experimental data showed close agreement with simulated injections delivered at the wound border (*r* = 5 mm) on day 0, but experimental data that included injections delivered on day 3 showed closer agreement to simulated injections delivered at *r* = 1 mm for later time points. Model simulations of an injection at the wound border (*r* = 5 mm) on day 3 show no difference compared to a vehicle simulation, suggesting that the model may underestimate the ability of the mRNA to penetrate the wound space and promote an increase in capillary tips at the healing wound border. We explore the impacts of altering the diffusivity of mRNA on wound healing in the next section. Furthermore, many of these simulations predict a very dramatic or nearly instantaneous acceleration of wound closure which may not be physiologically probable and can likely be attributed to the limited spatially resolved data available to fit parameters related to mRNA diffusion and VEGF-A generation rate. Since wound area is not a direct output of the PDE model, this instantaneous healing could also be an indication that blood vessel density may not correspond directly to wound area as quantified visually in an experimental model. However, the predicted relative differences in the rate of wound closure for injections delivered at various locations in the wound space remains an important finding.

### The Diffusivity of mRNA Affects Its Ability to Promote Wound Healing

Due to the limited availability of data regarding the spatial distribution of AZD8601 in the wound bed, we performed simulations with perturbations to the diffusion coefficient of mRNA to understand the impact of this spatial parameter on wound healing. This provides some insight about the effective diffusion length scale of AZD8601, but also provides an opportunity to apply this model to other drugs or methods of drug delivery with varied diffusion profiles.

**Figure 9:**
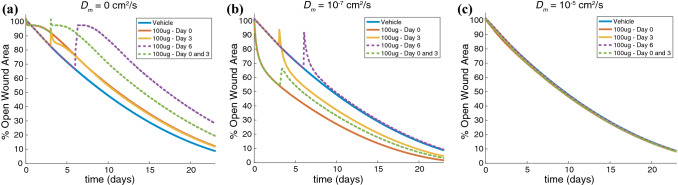
Diffusivity of mRNA impacts the rate of wound healing. Model predictions are displayed for a simulated injection of 100 *μ*g AZD8601 at the wound border (*r* = 5 mm) on day 0, day 3, day 6, or days 0 and 3 for a mRNA diffusion coefficient (*D*_*m*_) of (**a**) 0 cm^2^/s, (**b**) 10^−7^ cm^2^/s, and (**c**) 10^−5^ cm^2^/s.

We first performed model simulations with an mRNA diffusion coefficient (*D*_m_) of 0 cm^2^/s, which assumes that mRNA delivered at the wound border does not diffuse and is translated to VEGF-A only at the border. VEGF-A that is generated at the border due to the injection can diffuse some distance into the wound space as determined by the parameters of the chemoattractant equation (Eq. 23). For all simulated injection times (day 0, day 3, day 6, or day 0 and 3) this model predicted a delay in wound healing compared to a simulation with vehicle injection (Fig. 9a). A high concentration of VEGF-A is produced at the wound border, which causes an accumulation of capillary tips and blood vessels at the wound border (Supplemental Figure 1). This model implementation suggests that a drug intervention with restricted diffusion results in an accumulation of VEGF-A at the initial wound border, inhibiting normal capillary tip migration towards the center of the wound and thereby slowing the rate of wound healing.

Next, we performed model simulations with *D*_m_ = 10^−7^ cm^2^/s, two orders of magnitude higher than the literature reported values of intracellular mRNA diffusion used for simulations in Figs. 6, 7, 8. In this model implementation, an injection at day 0 is predicted to accelerate time to 50% wound closure by 5.5 days compared to a vehicle injection. When injections are delivered on both days 0 and 3, the second injection on day 3 causes some delay in wound closure, but accelerates time to 50% wound closure by 3.6 days compared to a vehicle injection. A single injection delivered on day 3, or day 6 causes an initial regression of wound area, but the day 3 injection is able to promote an acceleration in time to 50% wound closure of 2.6 days, while the injection at day 6 results in a time to 50% wound closure that is similar to that of a vehicle injection (Fig. 9b). Injections of AZD8601 create a gradient of VEGF-A inside the wound border, increasing the density of capillary tips and blood vessels at this location. The second injection at day 3 causes a temporary regression of capillary tips and blood vessels towards the wound border (Supplemental Figure 2). This model implementation demonstrates a better qualitative fit to the trends seen in the murine model of wound healing, where injections at day 0, day 3, or days 0 and 3 all promote an acceleration in wound healing compared to vehicle control groups.

Lastly, simulations with *D*_m_ = 10^−5^ cm^2^/s assumes that mRNA delivered by injections of AZD8601 at the wound border diffuses rapidly throughout the wound space. In this model implementation all simulated injection times (day 0, day 3, day 6, or days 0 and 3) showed no notable difference in rate of wound healing compared with a vehicle injection (Fig. 9c). In this scenario mRNA diffuses rapidly throughout the wound space, but is also lost more rapidly at the wound border to the surrounding tissue. The short-lived mRNA in the wound space increases VEGF-A concentration modestly throughout the wound space, but does not accelerate the migration of capillary tips or blood vessels towards the center of the wound (Supplemental Figure 3). All of these model implementations, however, assume that diffusivity of mRNA in the tissue surrounding the wound border is equivalent to the diffusivity in the wound bed. It is unlikely that the mRNA diffuses as rapidly in the surrounding tissue due to increased cellularity and density of extracellular matrix, which is discussed in more detail later.

## Discussion

We hypothesized that the location of proangiogenic drug delivery and the ensuing spatial gradients of growth factors would be a critical factor in the acceleration of diabetic wound healing. To investigate this hypothesis, we adapted a mechanistic mathematical model by Pettet *et al*.^29^ and coupled it with pharmacokinetic and pharmacodynamic models of a modified mRNA (AZD8601) designed to enhance VEGF-A protein expression in the skin, to predict how spatial delivery of this drug affects diabetic wound healing. We converted this published model to a cylindrical coordinate system to more accurately reflect the geometry of the circular wounds in our experimental model. We then deployed a sensitivity analysis to identify parameters appropriate for model fitting in order to calibrate the basal rate of wound healing in our computational model to a murine model of diabetic wound healing. We coupled a PDE that describes the spatiotemporal distribution of mRNA in the wound to the equation for chemoattractant, such that mRNA delivered through injections of AZD8601 at specified times and locations produced VEGF-A concentrations consistent with previous experimental measurements. We then used the model to simulate injections of AZD8601 at various times and locations throughout the wound area. Our model made predictions about how the location of drug delivery, combined with timing of delivery, affected wound healing rates and suggests that wound healing acceleration can be best achieved by repeatedly administering drug injections at a location 1–2 mm inside the healed wound border.

There are numerous challenges associated with evaluating the bioactivity of developmental pharmaceuticals in pre-clinical studies. In addition to the experimental variability inherent to animal model systems, studies are frequently carried out in laboratories in different locations and at different times, and experimental designs often vary from one study to the next, given the initial uncertainties in the dosage and dose scheduling. Experimental models are limited in the number of parameters that can feasibly be investigated (i.e., size of wound, dose of drug, route of drug delivery, timing of delivery, etc.), which often leads to arbitrary decisions about establishing parameters for experimental design. Computational models can be leveraged to conduct high-throughput variations in model parameters in order to identify those parameters that may be most consequential, and thus computational models can aid the design of pre-clinical and clinical studies. Previous computational models of wound healing have used differential equation-based methods to simulate the interactions between growth factors, cell populations, and ECM components. They have evaluated how different treatment protocols (timing and frequency of application) using commercially available engineered skin substitutes (Apligraf™ and Dermagraft™) impact healing,^40^ as well as other experimental treatments for diabetic ulcers, such as hyperbaric oxygen therapy.^11^ In other fields of study, such as cancer therapeutics, mathematical and computational models have been used more extensively to design complicated drug dosing schedules for pre-clinical^9^,^16^ and clinical trials.^15^

We generally note that models of different spatial and temporal scales and complexity are useful as complementary views of the same system and can be used to investigate phenomenon across biological, spatial, and temporal scales. We have recently reported an empirical pharmacokinetic and pharmacodynamic model of AZD8601 in wound healing.^1^ This previously published model captures wound healing dynamics at both the individual and the population level using a NLME modeling approach. Since the Almquist model is largely empirical, it may be difficult to interpret the biological consequences of model parameters. In comparison to the empirical NLME model, the advantage of the mechanistic model presented here is that it represents known biological processes, making model interpretation easy and facilitating future model revisions and extensions. For example, the mechanistic model is likely suitable for expansion to more than one growth factor at the same time, i.e., combination therapies. One limitation of the presented mechanistic model is that averaging of experimental measurements of wound area from a system with nonlinear dynamics may be inappropriate, whereas this problem is inherently addressed in the NLME approach of the empirical model which represents the wound healing dynamics on both the individual and the population levels. Another limitation of the model presented here is that we assume radial symmetry in the distribution of mRNA due to the computational complexity of a 2-D finite volume approach, despite the fact that injections in the animal model were delivered at four discrete locations separated by 90 degrees around the wound edge (see Fig. 1a).

Existing data for the experimental wound healing model is sparse, both with respect to the number of dependent variables that are currently accessible for measurement and the number of data-points in individual time and spatial series. Due to the lack of available spatially resolved data related to AZD8601 diffusion and clearance, we assumed a diffusion coefficient reported in literature for other mRNAs and a clearance rate by the vasculature comparable to that of protein. However, the diffusion coefficients reported in literature are representative of intracellular diffusion of mRNA and it is unclear what the effective length scale for extracellular diffusion would be especially in the setting of a cutaneous wound where there is likely less hindrance of diffusion due to increased matrix pore size and lack of, or remodeling extracellular matrix components.^24^,^36^ The wound healing cascade creates an extracellular environment that is constantly changing due to edema, neovascularization, altered cell density, and collagen content that would likely impact solute absorption through a wound site based on the timing of drug delivery and the phase of wound healing.^8^

This model provides unique and valuable insights about the spatial parameters of drug delivery that can be applied not only to future pre-clinical and clinical studies with AZD8601, but can also be generalized to the design of other wound healing studies with proangiogenic drugs. For example, previous studies with murine models of diabetic wound healing have also tested the efficacy of a recombinant VEGF-A applied topically to the wound.^38^ The model presented here could be adapted to simulate VEGF-A production that occurs uniformly throughout the wound space, or is applied in a bolus at specified time points, to further explore this mode of drug delivery. Our model predicts that wound healing could be accelerated by delivering injections of AZD8601 at a location inside the border of the healed wound, which would imply delivering injections in the underlying skeletal muscle of the wound bed. AZD8601 is intended to be delivered intradermally, so it remains to be determined whether injections with AZD8601 would be able to produce similar levels of VEGF-A if delivered intramuscularly, but this conclusion can be generalized to any drug application that is able to promote VEGF-A concentration in the wound area. Furthermore, the model could be extended to include a region of healthy tissue beyond the wound border and to simulate drugs with other time courses of action—drugs encapsulated in nanoparticles with mechanisms for controlled release, for example.^33^,^39^

Our model makes a number of assumptions about diabetic wound healing as it occurs in the murine model. Wound healing involves a complex cascade of molecular signals and cell behaviors that are not explicitly accounted for in our model, including hemostasis, inflammation, and extracellular matrix remodeling. In the diabetic wound, many aspects of the wound healing process are altered, and our model does not include the direct effects of disease on capillary sprouting, such as alterations in microRNAs that regulate these phases of inflammation and wound healing.^26^ Previous computational models of chronic wound healing and ulcers have also described the mechanical cues that regulate cell-cell adhesion and migration,^20^,^34^ and key inflammatory mediators that contribute to ulcer formation.^45^,^46^ Furthermore, in order to make direct comparisons between the predictions of the PDE model and experimental measurements of wound area (which measured wound closure by the extent of re-epithelization), it was necessary to assume that the PDE model prediction of blood vessel density was appropriate for estimating the extent of re-epithelization. We argue this is a reasonable assumption given the fact that re-epithelization requires deposition of granulation tissue, which is predominantly comprised of neovessels. We note, however, the discrepancies between the model predicted wound areas and experimental measurements of wound area at early time points (Figs. 3 and 8). In our murine model of wound healing, many wounds increase in size up to day 3 due to the effect of initial wound recoil^4^, which is not currently captured in the mechanisms of this wound healing model. Furthermore, our model does not explicitly account for the time delay required for cells to uptake the mRNA and begin producing VEGF-A, which has been documented experimentally.^1^,^27^

Although this model should be regarded as highly simplistic given the complex nature of diabetic wound healing, we believe that it provides a useful representation of the pre-clinical model and the effect of spatial delivery of AZD8601 on wound healing. Given the reported disconnects between small animal models of diabetic wounds and the clinical scenario in patients,^25^ it would be beneficial to identify methods and approaches that accurately scale findings in murine wound experiments to patient wounds, enabling data from pre-clinical studies to inform clinical trials more effectively. It is enticing to ponder whether and to what extent mathematical and computational models like ours and others^45^ could assist in this endeavor; however, a number of challenges would first need to be overcome. First, diabetic wounds in patients, the most common of which are diabetic foot ulcers, are typically 1.5–5 times larger in diameter and 2–5 times deeper than the standard small animal wound models (e.g., murine, rat, and rabbit), which range from 5 to 10 mm in diameter and 1 to 2 mm in depth, depending on the age and species of the animal. The assumptions that our model makes regarding the geometry may not hold in deeper wounds that have larger radii. Furthermore, the time scale of delayed healing in patient wounds can be orders of magnitude larger than what is typically observed in pre-clinical models, which is on the order of days-to-weeks, depending on the initial wound size, location, and species.^44^ The validity of extrapolating our model predictions from the relatively rapidly healing murine wounds to the more slowly healing human wounds remains to be confirmed.

These caveats notwithstanding, our model makes the interesting and not necessarily intuitive predictions that: (1) modifying the location of delivery of AZD8601 at varying distances from the center of the wound can accelerate the rate of wound closure, (2) limited diffusion of mRNA resulting in a gradient of VEGF-A that is highest at the wound border can inhibit capillary tip migration towards the center of the wound and even cause regression of blood vessels and delay wound healing, and (3) significant increases in the diffusivity of mRNA results in more loss of the mRNA at the wound border which reduces its ability to accelerate wound healing. In patients, it is not uncommon for diabetic foot ulcers to persist indefinitely until the decision to amputate, so complete wound closure is often unachievable. Therefore, although the FDA currently views complete closure as the only acceptable endpoint for clinical trials, predicting time to partial closure (e.g., 25 or 50% wound closure) may be more clinically helpful, given that a small amount of healing can substantially reduce the risk of infection and mitigate bioburden (e.g., bacteria) in the wound. Upon further validation, these model predictions may be generalizable to patients and could impact clinical trial design and ultimately the use of this drug in the clinical care of wounds.

## Electronic supplementary material

Below is the link to the electronic supplementary materials.

Supplementary material 1 (DOCX 77700 kb)

Supplementary material 2 (DOCX 105 kb)
