# Supplementary material for: Mathematical Model Predicts that Acceleration of Diabetic Wound Healing is Dependent on Spatial Distribution of VEGF-A mRNA (AZD8601)
Source: Cell Mol Bioeng. 2021 Jun 15;14(4):321–38. doi: 10.1007/s12195-021-00678-9 (PMC8280265; doi:10.1007/s12195-021-00678-9)
Supplement: Supplementary file 1 — Supplementary material 1 (DOCX 77700 kb) [file 12195_2021_678_MOESM1_ESM.docx]

**Supplemental Figures**

**Figure S1. Restricted diffusion of mRNA increases concentration of VEGF-A and density of capillary tips and blood vessels at the wound border following injections of 100 μg AZD8601 on days 0 and 3.** Heat maps in polar coordinates of the solutions for the governing equations: **(A)** chemoattractant concentration (*a*), **(B)** sprouting capillary-tip density (*n*), and **(C)** blood vessel density (*b*) for a simulation with restricted diffusion of mRNA (*D_m_* = 0 cm^2^/s) and repeated injections of 100 μg AZD8601 on days 0 and 3 (indicated by arrows). Radial coordinate corresponds to radius of the wound (0 - 5 mm), $\theta$coordinate corresponds to time (0 - 10 days), and color bar indicates value of the corresponding solution. **(D-F)** 2-D snapshots of the heatmaps in C-E at t = 0, 1, 2, 3, 4, 5, 6 and 7 days at the wound border (r = 3 - 5 mm).

**Figure S2. Moderate diffusion of mRNA creates gradient of VEGF-A inside the wound border following injections of 100 μg AZD8601 on days 0 and 3.** Heat maps in polar coordinates of the solutions for the governing equations: **(A)** chemoattractant concentration (*a*), **(B)** sprouting capillary-tip density (*n*), and **(C)** blood vessel density (*b*) for a simulation with moderate diffusion of mRNA (*D_m_* = 10^-7^ cm^2^/s) and repeated injections of 100 μg AZD8601 on days 0 and 3 (indicated by arrows). Radial coordinate corresponds to radius of the wound (0 - 5 mm), $\theta$coordinate corresponds to time (0 - 10 days), and color bar indicates value of the corresponding solution. **(D-F)** 2-D snapshots of the heatmaps in C-E at t = 0, 1, 2, 3, 4, 5, 6 and 7 days at the wound border (r = 3 - 5 mm).

**Figure S3. Rapid diffusion of mRNA produces modest, but short-lived increase in concentration of VEGF-A throughout the wound area following injections of 100 μg AZD8601 on days 0 and 3.** Heat maps in polar coordinates of the solutions for the governing equations: **(A)** chemoattractant concentration (*a*), **(B)** sprouting capillary-tip density (*n*), and **(C)** blood vessel density (*b*) for a simulation with rapid diffusion of mRNA (*D_m_* = 10^-5^ cm^2^/s) and repeated injections of 100 μg AZD8601 on days 0 and 3 (indicated by arrows). Radial coordinate corresponds to radius of the wound (0 - 5 mm), $\theta$coordinate corresponds to time (0 - 10 days), and color bar indicates value of the corresponding solution. **(D-F)** 2-D snapshots of the heatmaps in C-E at t = 0, 1, 2, 3, 4, 5, 6 and 7 days at the wound border (r = 3 - 5 mm).
