# Supplementary material for: Mathematical Model Predicts that Acceleration of Diabetic Wound Healing is Dependent on Spatial Distribution of VEGF-A mRNA (AZD8601)
Source: Cell Mol Bioeng. 2021 Jun 15;14(4):321–38. doi: 10.1007/s12195-021-00678-9 (PMC8280265; doi:10.1007/s12195-021-00678-9)
Supplement: Supplementary file 2 — Supplementary material 2 (DOCX 105 kb) [file 12195_2021_678_MOESM2_ESM.docx]

**Supplemental Methods**

[Section S1: Derivation of Governing Equations in Cylindrical Coordinates 1](#_Toc67473213)

[Section S2: Conversion of Initial and Boundary Conditions 4](#_Toc67473214)

[Section S3: Discretization Method Using Finite Differences 6](#_Toc67473215)

Section S1: Derivation of Governing Equations in Cylindrical Coordinates

The dimensionless model equations (Eqs. 1-3) described in Pettet *et al*., *Mathematical Biosciences* 1996, were converted from Cartesian coordinates to cylindrical coordinates by first defining the wound as a circle of radius R and rewriting the Cartesian form of the generalized, dimensionless governing equations (described in Pettet et al.) in general form (Eqs. 4-6), followed by evaluating the general forms in cylindrical coordinates. The conversion of the initial and boundary conditions to cylindrical coordinates is described in Section S2.

The dimensionless governing equations in 1-D Cartesian coordinates, as defined in Pettet et al., are

$\frac{\partial n}{\partial t}=\mu_{n}\frac{\partial^{2}n}{\partial x^{2}}-\chi\frac{\partial}{\partial x}\left(n\frac{\partial a}{\partial x}\right)+\lambda_{1}ab-\lambda_{2}n-\lambda_{0}n^{2}$ (1)

$\frac{\partial a}{\partial t}=\frac{\partial^{2}a}{\partial x^{2}}+\frac{\lambda_{4}}{2}\left[1+\tanh\left(\frac{1-b}{\delta}\right)\right]-(\lambda_{4}+\lambda_{5}b)a$ (2)

$\frac{\partial b}{\partial t}=\mu_{b}\frac{\partial}{\partial x}\left(n\frac{\partial b}{\partial x}\right)-\mu_{x}\frac{\partial n}{\partial x}+\chi n\frac{\partial a}{\partial x}$ (3)

These equations can be written more generally as

$\frac{\partial n}{\partial t}=-\nabla\bullet\text{J}_{\text{n}}+f_{n}(n,a,b,\nabla n,\nabla a,\nabla b)$ (4)

$\frac{\partial a}{\partial t}=-\nabla\bullet\text{J}_{\text{a}}+f_{a}(n,a,b,\nabla n,\nabla a,\nabla b)$ (5)

$\frac{\partial b}{\partial t}=-\nabla\bullet\text{J}_{\text{b}}+f_{b}(n,a,b,\nabla n,\nabla a,\nabla b)$ (6)

where **Jn**, **Ja**, and **Jb** are the dimensionless fluxes of capillary tips, chemoattractant, and blood vessels, respectively, and *fn*, *fa*, and *fb*are dimensionless kinetic quantities. Note that the $\nabla$ operator is also dimensionless.

Turning to the general governing equation for the capillary tip density, *n*, the dimensionless tip flux **Jn** in 1-D rectangular coordinates is given by

$\text{J}_{\text{n}}=-\mu_{n}\left[\frac{\partial n}{\partial x}+\chi n\frac{\partial a}{\partial x}\right]\text{e}_{\text{x}}$ (7)

where **ex** is the x-direction unit vector. Eq. 7 can be rewritten more generally as

$\text{J}_{\text{n}}=-u_{n}\nabla n+\chi n\nabla a$ (8)

Substituting Eq. 8 into Eq. 4 and expanding each term results in the following:

$\frac{\partial n}{\partial t}=-\nabla\bullet(-\mu_{n}\nabla n+\chi n\nabla a)+f_{n}$ (9)

$\frac{\partial n}{\partial t}=\mu_{n}\nabla^{2}n-\chi\nabla\bullet(n\nabla a)+f_{n}$ (10)

$\frac{\partial n}{\partial t}=\mu_{n}\nabla^{2}n-\chi\left[n(\nabla^{2}a)+\nabla a(\nabla n)\right]+f_{n}$ (11)

Evaluating the *r*-component of the differential operators in cylindrical coordinates gives the desired dimensionless governing equation describing the radial distribution of the capillary tip density *n* within the wound:

$\frac{\partial n}{\partial t}=\mu_{n}\frac{1}{r}\frac{\partial}{\partial r}\left(r\frac{\partial n}{\partial r}\right)-\chi n\frac{1}{r}\frac{\partial}{\partial r}\left(r\frac{\partial a}{\partial r}\right)-\chi\frac{\partial a}{\partial r}\frac{\partial n}{\partial r}+\lambda_{1}ab-\lambda_{2}n-\lambda_{0}n^{2}$ (12)

Turning to the chemoattractant, *a*, the dimensionless flux in 1-D Cartesian coordinates and in general differential form, respectively, is given by

$\text{J}_{\text{a}}=-\frac{\partial a}{\partial x}\text{e}_{\text{x}}$ (13)

$\text{J}_{\text{a}}=-\nabla a$ (14)

Substituting Eq. 14 into Eq. 4 yields

$\frac{\partial a}{\partial t}=\nabla^{2}a+f_{a}$ (15)

which is further developed by evaluating the *r*-component of $\nabla^{2}$ in cylindrical coordinates and expanding the kinetic term *fa* to give the governing equation of chemoattractant in the *r*-direction:

$\frac{\partial a}{\partial t}=\frac{1}{r}\frac{\partial}{\partial r}\left(r\frac{\partial a}{\partial r}\right)+\frac{\lambda_{4}}{2}\left[1+\tanh\left(\frac{1-b}{\delta}\right)\right]-(\lambda_{4}+\lambda_{5}b)a$ (16)

Applying the same approach for the blood vessel density *b* results in the following:

$\text{J}_{\text{b}}=-\mu_{b}n\nabla b$ (17)

$\frac{\partial b}{\partial t}=\mu_{n}\left[n\nabla^{2}b+\nabla b(\nabla n)\right]+f_{b}$ (18)

$\frac{\partial b}{\partial t}=\mu_{n}\left[n\frac{1}{r}\frac{\partial}{\partial r}\left(r\frac{\partial b}{\partial r}\right)+\frac{\partial b}{\partial r}\frac{\partial n}{\partial r}\right]-\mu_{n}\frac{\partial n}{\partial r}+\chi n\frac{\partial a}{\partial r}$ (19)

Thus, the dimensionless forms of the governing equations in cylindrical coordinates are given by Equations 12, 16, and 19 for variables *n*, *a*, and *b*, respectively.

*Definitions of Dimensionless Quantities*

All dimensionless parameters and quantities that appear in Eqs. 12, 16, and 19 retain their original definitions and forms from Pettet et al. (found between Equations 9 and 10 in Pettet et al.) except that, instead of scaling *x* by *L* (the wound half-length in Pettet et al.), we scale the radial coordinate *r* by *R*, the wound radius in our system. The characteristic length (*L*) in the original model described by Pettet et al. was *L* = 2.5 mm. The radius of the wound (*R*) described by our model in cylindrical coordinates is *R =* 5 mm. Accordingly, *L* is replaced with *R* according to *R =* 2*L* in the definitions of all dimensionless parameters and quantities, where applicable. The time coordinate *t* is made dimensionless in our model by scaling it by the quantity *D/R2*, where *D* is the diffusivity of chemoattractant. The physical interpretations of all common parameters in our model are the same as those described by Pettet et al. Descriptions and values for these parameters are given in Table 3.

Section S2: Conversion of Initial and Boundary Conditions

Converting the coordinate system from Cartesian coordinates to cylindrical coordinates in *r* requires that the initial and boundary conditions be rewritten accordingly with the wound center defined at *r = 0* (instead of *x = L*) and the wound edge at *r = R* (instead of *x = 0*), where *R* is the dimensional wound radius. For derivatives defined at the wound boundaries, *x* is simply substituted for *r* in the conversion to cylindrical coordinates. After converting to dimensionless variables, the wound center in Cartesian coordinates at *x = L* becomes *x =* 1 after scaling *x* by *L*; similarly for *r* in cylindrical coordinates, the wound edge at *r* = *R* becomes *r =* 1 after scaling *r* by *R*.

Dimensionless Cartesian boundary and initial conditions (from Pettet et al.)

The following are the initial and boundary conditions used in the wound healing model described by Pettet et al. It is assumed that the wound margin has penetrated an initial distance $\tilde{x}$ into the wound such that the open wound has the dimensionless width 1 − $\tilde{x}$.

*Initial conditions:*

$n\left( x,0 \right)= \left\{ \begin{aligned} \frac{\hat{n}}{\tilde{x}^{3}}\left( x-\tilde{x} \right)\left( 2x^{2}-\tilde{x}x-\tilde{x}^{2} \right), 0\leq x\leq\tilde{x} \\ 0, \tilde{x}<x\leq1 \end{aligned} \right.$ (20)

$a\left( x,0 \right)=0, 0\leq x\leq1$ (21)

$b\left( x,0 \right)= \left\{ \begin{aligned} \left( \frac{\hat{b}-\tilde{b}}{\tilde{x}^{3}} \right)\left( x-\tilde{x} \right)\left( 2x^{2}-\tilde{x}x-\tilde{x}^{2} \right)+b, 0\leq x\leq\tilde{x} \\ \tilde{b}, \tilde{x}<x\leq1 \end{aligned} \right.$ (22)

*Boundary conditions:*

| $n\left( 0,t \right)= \hat{n}e^{-\alpha t}$ (23) | $\frac{\partial n}{\partial x}\left( 1,t \right)=0$ (24) |
| --- | --- |
| $\frac{\partial a}{\partial x}\left( 0,t \right)= -\lambda_{7}a(0,t)\hat{b}$ (25) | $\frac{\partial a}{\partial x}\left( 1,t \right)=0$ (26) |
| $b\left( 0,t \right)= \hat{b}$ (27) | $\frac{\partial b}{\partial x}\left( 1,t \right)=0$ (28) |

Dimensionless cylindrical boundary and initial conditions

After converting to cylindrical coordinates, the above boundary and initial conditions take the forms listed below. It is again assumed that the wound margin has penetrated an initial distance *R* − $\tilde{r}$ such that the radius of the open wound is $\tilde{r}$.

*Initial conditions:*

$n\left( r,0 \right)= \left\{ \begin{aligned} \frac{\hat{n}}{\tilde{r}^{3}}\left( r-\tilde{r} \right)\left( 2r^{2}-\tilde{r}r-\tilde{r}^{2} \right), \tilde{r}<r\leq1 \\ 0, 0\leq r\leq\tilde{r} \end{aligned} \right.$ (29)

$a\left( r,0 \right)=0, 0\leq r\leq1$ (30)

$b\left( r,0 \right)= \left\{ \begin{aligned} \left( \frac{\hat{b}-\tilde{b}}{\tilde{r}^{3}} \right)\left( r-\tilde{r} \right)\left( 2r^{2}-\tilde{r}r-\tilde{r}^{2} \right)+b, \tilde{r}<r\leq1 \\ \tilde{b}, 0\leq r\leq\tilde{r} \end{aligned} \right.$ (31)

*Boundary conditions:*

| $\frac{\partial n}{\partial r}\left( 0,t \right)=0$ (32) | $n\left( 1,t \right)= \hat{n}e^{-\alpha t}$ (33) |
| --- | --- |
| $\frac{\partial a}{\partial r}\left( 0,t \right)=0$ (34) | $\frac{\partial a}{\partial r}\left( 1,t \right)= {-\lambda}_{7}a\left( 1,t \right)\hat{b}$ (35) |
| $\frac{\partial b}{\partial r}\left( 0,t \right)=0$ (36) | $b\left( 1,t \right)= \hat{b}$ (37) |

Section S3: Discretization Method Using Finite Differences

*Forward difference method for first-order time derivatives.* First-order derivatives in time were discretized using an explicit forward finite difference formula, where *h* = *ti+1­* – *ti* is the time step size between two discretized time points, *C* represents the concentration of an arbitrary field variable, and *λ* is an arbitrary constant whose definition depends on the specific field variable being evaluated:

$\frac{\partial C}{\partial t}=\lambda C$ (38)

$\frac{C_{j,i+1}-C_{j,i}}{h}=\lambda C_{j,i}$ (39)

*Central difference method for first-order spatial derivatives.* First-order derivatives in space were discretized using an explicit first-order central finite difference formula, where *k* is the spatial step size in *r* between two discretized spatial nodes (*k = rj+1* – *rj*), as follows for species *C*:

$\frac{\partial C}{\partial r}=\lambda C$ (40)

$\frac{C_{j+1,i}-C_{j-1,i}}{2k}=\lambda C_{j,i}$ (41)

*Central difference method for second-order spatial derivatives.* Second-order derivatives in space were discretized using an explicit second-order central finite difference formula:

$\frac{\partial^{2}C}{\partial r^{2}}=\lambda C$ (42)

$\frac{C_{j+1,i}-2C_{j,i}+C_{j-1,i}}{k^{2}}=\lambda C_{j,i}$ (43)

*Discretization of model equations in cylindrical coordinates.* Implementation of the finite difference schemes is illustrated with the following example of discretizing the governing equation and boundary conditions for the density of capillary tips, *n*. Evaluating the r-component of the differential operators in cylindrical coordinates, expanding the differentials, and discretizing the result yields

$\frac{\partial n}{\partial t}=\mu_{n}\left(\frac{1}{r}\frac{\partial n}{\partial r}+\frac{\partial^{2}n}{\partial r^{2}}\right)-\chi n\left(\frac{1}{r}\frac{\partial a}{\partial r}+\frac{\partial^{2}a}{\partial r^{2}}\right)-\chi\frac{\partial a}{\partial r}\frac{\partial n}{\partial r}+\lambda_{1}ab-\lambda_{2}n-\lambda_{0}n^{2}$ (44)

$\begin{matrix} \frac{n_{j,i+1}-n_{j,i}}{h}=\mu_{n}\left(\frac{1}{r_{j}}\frac{n_{j+1,i}-n_{j-1,i}}{2k}+\frac{n_{j+1,i}-2n_{j,i}+n_{j-1,i}}{k^{2}}\right)-\chi n\left(\frac{1}{r_{j}}\frac{a_{j+1,i}-a_{j-1,i}}{2k}+\frac{a_{j+1,i}-2a_{j,i}+a_{j-1,i}}{k^{2}}\right)- \\ \chi\left(\frac{a_{j+1,i}-a_{j-1,i}}{2k}\right)\left(\frac{n_{j+1,i}-n_{j-1,i}}{2k}\right)+\lambda_{1}a_{j,i}b_{j,i}-\lambda_{2}n_{j,i}-\lambda_{0}n_{j,i}^{2} \end{matrix}$ (45)

This discretized equation can be rearranged to solve for the dimensionless tip density *n* at time *ti+1*and spatial node *rj* (*nj,i+1*), given that values for *n* are known at spatial nodes rj-1, rj, and rj+1 at time point ti (represented by *nj-1,i*, *nj,i*, and *nj+1,i*, respectively) accordingly:

$\begin{matrix} n_{j,i+1}=\left\{\mu_{n}\left(\frac{1}{r_{j}}\frac{n_{j+1,i}-n_{j-1,i}}{2k}+\frac{n_{j+1,i}-2n_{j,i}+n_{j-1,i}}{k^{2}}\right)-\chi n\left(\frac{1}{r_{j}}\frac{a_{j+1,i}-a_{j-1,i}}{2k}+\frac{n_{j+1,i}-2a_{j,i}+a_{j-1,i}}{k^{2}}\right)\right.- \\ \left.\chi\left(\frac{a_{j+1,i}-a_{j-1,i}}{2k}\right)\left(\frac{n_{j+1,i}-n_{j-1,i}}{2k}\right)+\lambda_{1}a_{j,i}b_{j,i}-\lambda_{2}n_{j,i}-\lambda_{0}n_{j,i}^{2}\right\}h+n_{j,i} \end{matrix}$ (46)

*Discretized boundary conditions.* Discretization of Dirichlet boundary conditions requires relating the concentration of species of interest at time *ti* to the value prescribed by the condition, as follows:

$n(1,t)=\hat{n}e^{-\alpha t}$ (47)

$n_{j,i}=\hat{n}e^{\alpha t_{i}}$ (48)

where *j* corresponds to the spatial node at the wound edge.

No-flux Neumann boundary conditions at the wound center require that the concentration of a given species at the boundary node be set equal to the interior node immediately adjacent to it. Thus, at the wound center:

$\frac{\partial n}{\partial r}(0,t)=0$ (49)

$\frac{n_{j+1,i}-n_{j,i}}{k}=0$ (50)

$n_{j,i}=n_{j+1,i}$ (51)

where *j* in this case indicates the boundary node, located at the wound center.

Reactive Robin boundary conditions are similarly discretized and solved. Using the boundary condition for chemoattractant at the wound edge as an example:

$\frac{\partial a}{\partial r}(1,t)=-\lambda_{7}a(1,t)\hat{b}$ (52)

$\frac{a_{j+1,i}-a_{j,i}}{k}=-\lambda_{7}a_{j+1,i}\hat{b}$ (53)

$a_{j+1,i}=\frac{a_{j,i}}{1+k\lambda_{7}a_{j+1,i}\hat{b}}$ (54)

where *j+*1 in this case indicates the boundary node, located at the wound edge.

*Solutions to discretized model equations.* At a given time point, the partial differential equations for the field variables were discretized using the explicit finite difference method described above. The system of algebraic equations which results from discretizing over the wound space domain was then explicitly solved using the known concentrations of the field variables from the previous time point, followed by evaluating the discretized boundary conditions. The system of equations was then advanced to the next time point, and this process was repeated until the final time point was reached. The general method is summarized according to the following steps:

1. All partial differential equations were discretized using finite difference methods.
2. Concentrations of all field variables were defined for the initial time.
3. The model was advanced one time step, and the concentrations of all field variables were solved for in the bulk (i.e., everywhere except the wound center and wound edge boundaries).
4. At the same time step, the concentrations of all field variables at the wound center were solved for using the appropriate boundary conditions at *r =* 0.
5. At the same time step, the concentrations of all field variables at the wound edge were solved for using the appropriate boundary conditions at *r =* 1.

Steps 3-5 were repeated until the final time point was reached.
